# Complete Resolution of Frequent, Non-Specific Hypoglycemia Symptoms After Surgical Removal in a Patient With Pancreatic Tail Insulinoma: A Case Report

**DOI:** 10.7759/cureus.77662

**Published:** 2025-01-19

**Authors:** Fariz N Huda, Susanna H Hutajulu, Sofi Aresy, Raden B Pramono, Agus Barmawi, Auliya S Sumpono, Nurhuda H Setyawan

**Affiliations:** 1 Department of Internal Medicine, Faculty of Medicine, Public Health, and Nursing, Universitas Gadjah Mada/Dr. Sardjito General Hospital, Yogyakarta, IDN; 2 Division of Hematology and Medical Oncology, Department of Internal Medicine, Faculty of Medicine, Public Health, and Nursing, Universitas Gadjah Mada/Dr. Sardjito General Hospital, Yogyakarta, IDN; 3 Division of Endocrinology, Metabolic Disease, and Diabetes, Department of Internal Medicine, Faculty of Medicine, Public Health, and Nursing, Universitas Gadjah Mada/Dr. Sardjito General Hospital, Yogyakarta, IDN; 4 Division of Digestive Surgery, Department of Surgery, Faculty of Medicine, Public Health, and Nursing, Universitas Gadjah Mada/Dr. Sardjito General Hospital, Yogyakarta, IDN; 5 Department of Anatomical Pathology, Faculty of Medicine, Public Health, and Nursing, Universitas Gadjah Mada/Dr. Sardjito General Hospital, Yogyakarta, IDN; 6 Division of Radiology Diagnostic, Department of Radiology, Faculty of Medicine, Public Health, and Nursing, Universitas Gadjah Mada/Dr. Sardjito General Hospital, Yogyakarta, IDN

**Keywords:** case report, diagnostic, functional neuroendocrine tumor, hypoglycemia, insulinoma, pancreas

## Abstract

Insulinoma, the most common functional pancreatic neuroendocrine tumor, is a rare and typically benign tumor. However, excessive insulin secretion can result in life-threatening hypoglycemia, which is often misdiagnosed due to its nonspecific early symptoms, leading to a delayed diagnosis. Rigorous evaluation is required once insulinoma is suspected since the tumor might also be challenging to detect through imaging techniques. This case study explores insulinoma's diagnostic, localization, and management challenges. We present the case of a 41-year-old female patient who had multiple episodes of severe hypoglycemia for the past four years. A combination of biochemical and morphological examinations led to the identification of a localized pancreatic insulinoma. Subsequently, an open surgical procedure was performed, resulting in a successful removal of the insulinomas through enucleation and remarkable post-operative result. It is essential to consider insulinoma as a potential underlying cause of recurrent hypoglycemia in non-diabetic individuals. High awareness might significantly reduce the risk of delayed diagnosis of the disease, and successful surgical removal of the insulinoma not only alleviates hypoglycemic symptoms but also leads to a complete resolution of the condition, preventing life-threatening complications associated with untreated hypoglycemia.

## Introduction

Insulinoma, the most prevalent neoplasm among functional pancreatic neuroendocrine tumors (P-NETs), is a rare entity with an estimated incidence of 0.4 per 100,000 person-years [1]. Previous cohort studies have reported that insulinomas are most commonly diagnosed around the median age of 50 years, with a female predominance [1,2]. More than 90% of insulinomas are benign and most frequently present as sporadic (non-inherited) pancreatic islet cell tumors [3]. Their excessive insulin secretion can lead to significant clinical manifestations, typically characterized by Whipple’s triad, indicated by symptoms of neuroglycopenia occurring during episodes of hypoglycemia, which resolve with glucose correction. The timing of symptoms may occur not only during fasting but also as postprandial hypoglycemia, which can concurrently occur or even present as the sole manifestation of hypoglycemia [2].

Due to their nonspecific early clinical presentation, insulinomas are frequently misdiagnosed as other medical conditions. Up to 20% of patients with insulinoma are initially misdiagnosed with a neurological or psychiatric disorder before the correct diagnosis is established [1,4]. Detection through imaging techniques can also be challenging, with approximately 70% of insulinoma detected with combined transabdominal ultrasound and triple-phase spiral computed tomography (CT) of the pancreas, as shown in previous studies [2]. Surgical excision remains the treatment of choice and is typically curative [1]. However, the precise localization of the tumor can be particularly challenging, as some tumors may not be palpable during surgery [5]. Given the rarity and the diagnostic and surgical challenges associated with insulinoma, we present a case study that explores the diagnosis, localization, and management strategies for this rare condition.

## Case presentation

A 41-year-old Javanese woman presented to the hospital's emergency department with cold sweats and syncope that had occurred one hour prior to admission. On arrival, her blood glucose level was critically low at 16 mg/dL. Her consciousness improved after intravenous administration of 50 mL of 40% dextrose solution, and her blood glucose level increased to 100 mg/dL upon evaluation after treatment. She reported a four-year history of recurrent severe hypoglycemic episodes. Over those years, the patient managed symptoms of weakness, which occurred frequently, by consuming sugar water as an immediate response. In more severe episodes resulting in loss of consciousness, she was frequently brought to the emergency department for intravenous glucose administration. The patient had initially been diagnosed with adrenal insufficiency and was prescribed dexamethasone 0.5 mg once daily, a regimen she adhered to consistently until her presentation at our center. After prolonged steroid use and frequent sugar water consumption, the patient significantly gained weight of 18 kg, along with the development of a cushingoid appearance characterized by moon facies, striae, and central obesity over this period. Her family history was unremarkable. Physical examination revealed a healthy-appearing female with a body mass index (BMI) of 27.3 kg/m^2^. Whipple’s triad was confirmed, indicated by symptoms and signs of hypoglycemia, a documented low plasma glucose concentration of 16 mg/dL (normal range 80-120 mg/dL), and resolution of symptoms following glucose administration. Laboratory evaluations showed significantly elevated levels of C-peptide and insulin, measured at 12.370 ng/mL (normal range 1.1-4.4 ng/mL) and 113.40 μU/mL (normal range 2.6-24.9 μU/mL), respectively.

**Figure 1:**
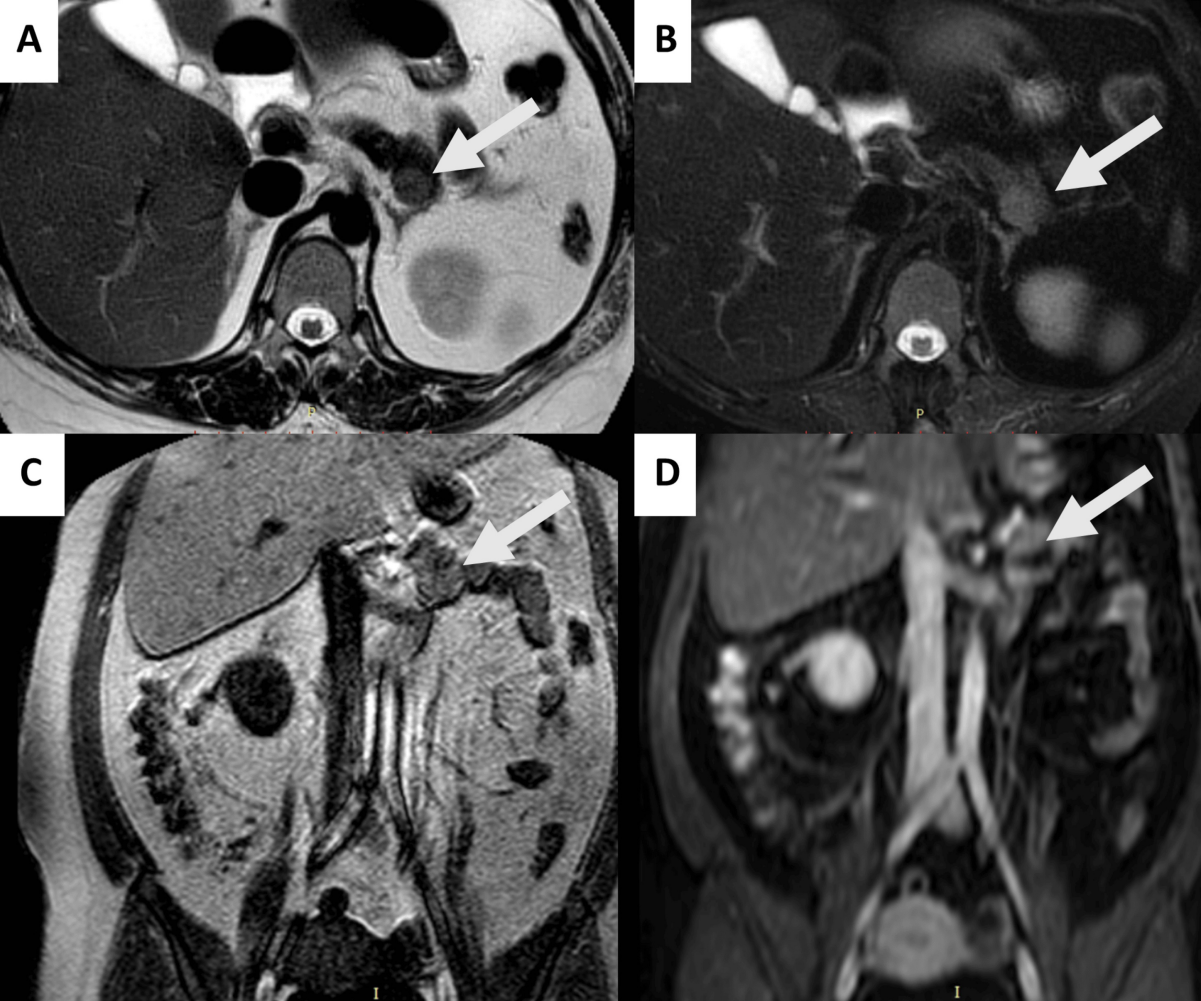
Abdominal MRI with intravenous contrast revealing a solid mass in the pancreatic tail (white arrow), which is round, well-defined, with regular margins, and measures 1.89 x 1.49 cm. The lesion appears slightly hyperintense compared to the surrounding normal pancreatic parenchyma on (A) axial T2-weighted image and (B) axial T2-weighted image with fat suppression. The lesion is isointense compared to the surrounding pancreatic parenchyma on (C) coronal T1-weighted image. No significant enhancement post-gadolinium contrast injection or infiltration into the adjacent structures is observed on (D) post-contrast coronal T1-weighted image.

Given the repeated history of hypoglycemia, the confirmation of Whipple's triad, and elevated fasting C-peptide and insulin levels, a diagnosis of insulinoma was strongly suspected. During further history-taking, the patient disclosed that she had previously been referred to a gastrointestinal surgeon at another healthcare facility due to a suspected diagnosis of insulinoma. She was advised to undergo further diagnostic evaluation and treatment but declined due to concerns and fear surrounding the possibility of surgical intervention. Abdominal magnetic resonance imaging (MRI) with intravenous contrast identified a solid lesion in the pancreatic tail, measuring 1.89 x 1.49 cm. The lesion appeared round, well-defined, and with regular margins. On axial T2-weighted images, the mass demonstrated slightly hyperintense signal intensity compared to the surrounding normal pancreatic parenchyma, which was more pronounced on T2-weighted images with fat suppression. In coronal T1-weighted images, the lesion appeared isointense relative to the pancreatic parenchyma. Post-contrast coronal T1-weighted imaging revealed no significant enhancement or evidence of infiltration into adjacent structures. These findings, combined with the patient’s clinical presentation, strongly suggested an insulinoma (Figure 1).

**Figure 2:**
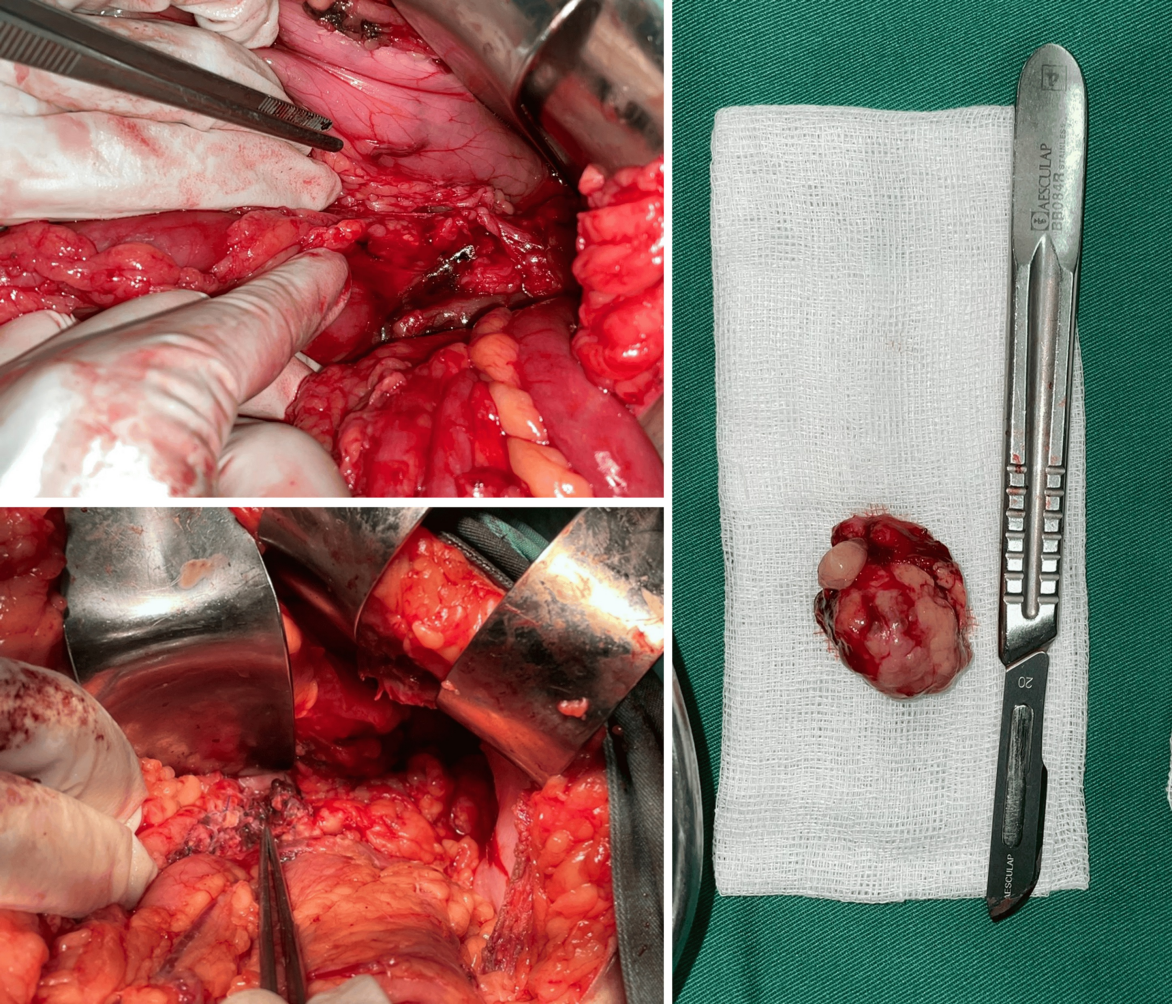
(A) The operative view revealed a cystic mass located in the pancreatic tail (indicated by the green arrow). (B) The gross resected specimen showed the enucleated mass from the pancreatic tail with a solid and partially trabecular structure. (C) The post-operative view displayed the resected tumor.

The patient was subsequently referred to the gastrointestinal surgery department and underwent exploratory laparotomy. Tumor enucleation revealed a mass measuring 3.5 x 2.5 x 1.5 cm located in the distal one-third of the pancreas (Figure 2).

**Figure 3:**
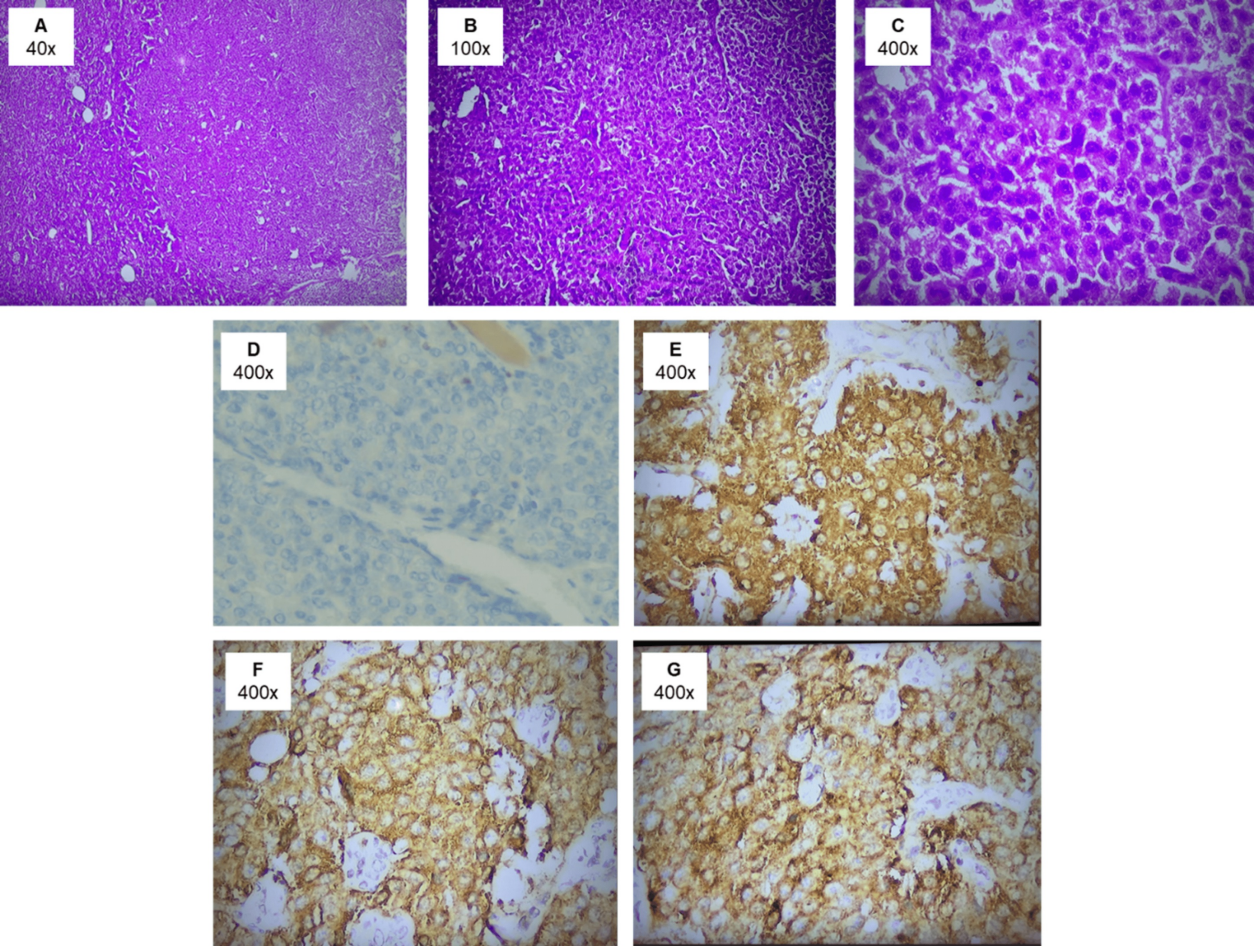
(A-C) Histopathological images of the tumor cells at low, medium, and high magnifications, respectively, stained with hematoxylin-eosin. The tumor cells exhibit mild pleomorphism, minimal cytoplasm, and nuclei that are round, oval, and hyperchromatic. The stroma displays vascular dilation. (D) A high-magnification image showing the Ki-67 proliferation index, estimated at 5%. (E-G) Immunohistochemistry staining results (all at high magnification): (E) tumor cells showing positivity for synaptophysin, (F) tumor cells showing positivity for chromogranin, and (G) tumor cells showing positivity for insulin.

Post-operative histopathological analysis of the specimen revealed a tumor with a solid and partially trabecular architecture. The tumor cells exhibited mild pleomorphism with minimal cytoplasm, and the nuclei appeared round, oval, and hyperchromatic. Additionally, the stroma showed evidence of vascular dilation. Immunohistochemistry staining was positive for chromogranin, synaptophysin, and insulin, displaying strong cytoplasmic intensity in the majority of tumor cells. The Ki-67 proliferation index was estimated to be 5%. Based on the functional status and biological behavior, a definitive diagnosis of a well-differentiated (grade 2) P-NET was established (Figure 3).

**Figure 4:**
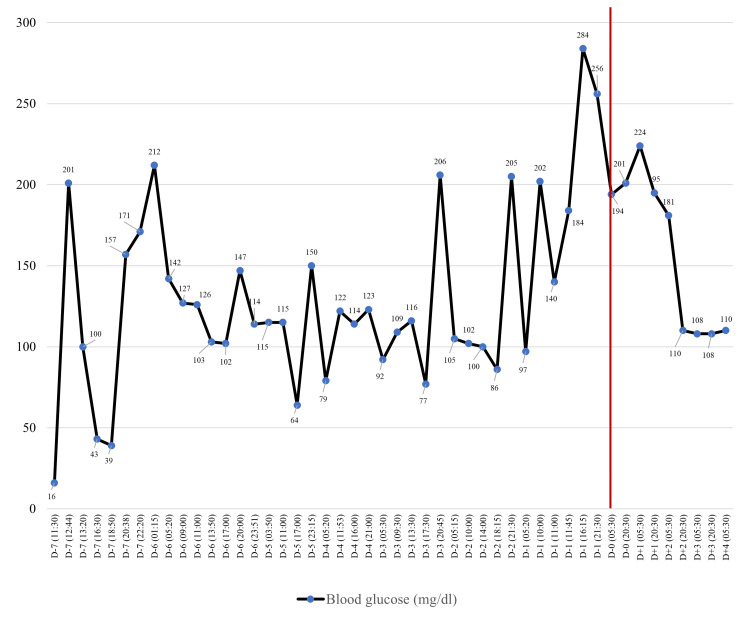
Blood glucose levels from 7 days before surgery (D–7) to 4 days after surgery (D+4). The red line indicates the day of the surgery.

The postoperative clinical result was remarkable, leading to an immediate and sustained normalization of plasma glucose levels. The C-peptide and insulin levels after surgery showed a significant reduction, measured at 1.98 ng/mL (normal range 1.1-4.4 ng/mL) and 5.3 μU/mL (normal range 2.6-24.9 μU/mL), respectively. The levels of blood glucose before surgery and stabilization after surgery are depicted in Figure 4. Steroids were administered as part of the perioperative management and were gradually tapped by the endocrinologist after the surgery.

The patient continued to remain symptom-free from hypoglycemic signs and symptoms throughout the follow-up period, ultimately achieving full recovery following surgery. During the one-year follow-up, the patient continued to be symptom-free.

## Discussion

Among functional P-NETs, insulinomas are the most prevalent, representing approximately 20.9% of cases [6]. Typically, insulinomas are benign and well-differentiated with a favorable prognosis, although approximately 5.8% of insulinomas exhibit malignant potential [1]. Early manifestations of P-NETs are frequently linked to functional syndromes due to the secretion of bioactive substances. However, in cases in which functional hormonal syndromes are not apparent, symptoms are often associated with tumor size, local invasion, or distant metastasis [7]. The accurate diagnosis of insulinoma primarily depends on clinical presentation and laboratory findings. In the evaluation of a patient with hyperinsulinemic hypoglycemia, it is imperative to establish Whipple's triad as an initial step. In our case, the patient presented with altered consciousness and cold sweats. Patients should present with symptoms of hypoglycemia, which may include autonomic symptoms (such as tremor, раlрitаtiοոѕ, anxiety or arousal, sweating, hunger, and paresthesia) or neuroglycopenic symptoms (such as dizziness, weakness, drowsiness, cognitive impairment, behavioral changes, and altered consciousness) [8,9], and signs such as diaphoresis, pallor, and increased heart rate [10]. The symptoms of insulinoma were typically progressive, triggered by fasting, and, at worst, happened daily or weekly. In a prior study, 77% of patients experienced autonomic symptoms and 96% had neuroglycopenic symptoms [11]. Weight gain is also seen in some patients, arising from either overeating to alleviate hypoglycemic symptoms or from the anabolic effects of excessive insulin production, which are often triggered by fasting or physical activity [12]. A previous study also highlighted that patients with insulinoma had increased weight, with one-third gained weight more than 10 kg [11]. Alongside concurrent low blood glucose levels (typically less than 50 mg/dL), these symptoms should resolve upon glucose intake or correction of low blood glucose levels [13].

In our case, the diagnosis of insulinoma was significantly delayed, spanning several years before the correct identification was made. Chronic hypoglycemia due to insulinoma often manifests as neuroglycopenic symptoms, which can easily be overlooked or misdiagnosed. Furthermore, with prolonged and recurrent hypoglycemia, neurogenic symptoms tend to diminish, while nonspecific neuroglycopenic symptoms become predominant [14], which might explain the delayed diagnosis and prolonged periods of untreated hypoglycemia in our case. Hypoglycemia symptoms might also be misattributed to symptoms of neurological, psychiatric, or even cardiac system disorder, with a previous study identifying that up to 20% of patients with insulinoma are initially misdiagnosed [1,4]. A previous study found that the median duration of symptoms before diagnosis was less than 1.5 years, although a few patients may have experienced symptoms for decades [1]; in our case, it took four years for the patient to receive appropriate diagnosis and treatment. Therefore, clinicians should maintain a high suspicion of insulinoma in patients presenting with recurrent hypoglycemia to prevent diagnostic delays and mitigate associated risks. Another factor contributing to the delay in diagnosis was patient-related. The patient had a history of recurrent hypoglycemic episodes but delayed further investigation and treatment due to fear of surgery. Such delays also prolong the timely diagnosis of insulinoma. Therefore, clinicians should also clearly communicate the importance of timely diagnosis and treatment, address any concerns about procedures, offer psychological support or counseling if needed, and educate patients on the risks of delaying diagnosis, emphasizing the potential for life-threatening conditions and the need for early intervention.

Once Whipple's triad is confirmed, a supervised 72-hour fast is typically performed, considered the gold standard test for the diagnosis of insulinoma [15], which involves measuring the plasma glucose, insulin, C-peptide, and proinsulin levels every 6 hours until the plasma glucose reaches 60 mg/dL or lower, at which point the testing interval is reduced to every 1-2 hours. The fast is concluded when the plasma glucose level falls to 45 mg/dL or lower and the patient exhibits signs and symptoms of hypoglycemia. Elevated insulin (≥6 μU/mL) and C-peptide (≥0.2 nmol/L) levels in the presence of hypoglycemia are highly suggestive of insulinoma, following the exclusion of other causes such as exogenous insulin or hypoglycemic agents [16]. Given the extended duration of this test and the need for hospitalization, which can be challenging and uncomfortable for patients while also demanding significant human and financial resources, recent studies have explored shorter fasting protocols, such as the 48-hour fast [17] and overnight fasting [18], as a potential first-line diagnostic approach for insulinoma. In our case, insulin and C-peptide levels were measured during a hypoglycemic episode upon admission. Due to the aforementioned challenges, we were unable to perform the 72-hour fasting protocol. Nonetheless, the insulin and C-peptide levels observed still indicate endogenous insulin production [19]. Hypoglycemia can also be managed with glucocorticoids, but long-term use of these medications has a high risk of adverse effects, including weight gain, neuropsychiatric disorders, hypertension, and secondary opportunistic infections.

In hypoglycemic cases where organic hyperinsulinism is confirmed in symptomatic patients, imaging becomes imperative for locating the tumor and guiding subsequent surgical management [20]. Imaging plays a pivotal role in the diagnosis, localization, and management of insulinomas. The choice of imaging technique is often guided by the clinical presentation, lesion size, and institutional expertise. Various techniques are available, each offering unique advantages and limitations. Ultrasound, particularly endoscopic ultrasound (EUS) with fine needle aspiration (FNA), provides high-resolution images for small lesions and is especially useful for tumors located deep within the pancreas. CT and MRI are the most commonly employed cross-sectional imaging modalities for insulinoma localization. CT, especially multiphase or dynamic contrast-enhanced imaging, can detect small lesions due to the hypervascular nature of insulinomas, which often exhibit intense enhancement during the arterial or pancreatic parenchymal phase. MRI offers superior soft-tissue contrast and can differentiate between the lesion and surrounding pancreatic tissue, with typical findings including hyperintensity on T2-weighted images and variably strong post-contrast enhancement [21,22]. Observed using MRI with intravenous contrast, the lesion size of our case is typical as the measure of insulinomas are mostly less than 2 cm [23]. On T2-weighted and fat-suppressed T2-weighted images, the mass shows hyperintense signal intensity, and on T1-weighted and post-contrast coronal T1-weighted imaging, the lesion appears isointense relative to the pancreatic parenchyma, which is a common finding in insulinomas [24]. Furthermore, the absence of infiltration suggests that the tumor is localized and potentially resected.

Advanced imaging modalities, such as 68Ga-DOTATATE positron emission tomography/computed tomography (PET/CT) and fluorine-18-L-dihydroxyphenylalanine (18-F-DOPA) PET, provide highly sensitive and specific tumor localization by leveraging the neuroendocrine tumor’s ability to decarboxylate amine precursors or express somatostatin receptors. These molecular imaging techniques are particularly valuable in cases where conventional imaging modalities fail to detect the tumor or when multiple lesions are suspected. Additionally, intra-arterial stimulation with venous sampling (ASVS) and arteriography remain useful in select cases for functional localization when imaging findings are inconclusive. In the context of surgical planning, imaging is critical not only for confirming the presence of the tumor but also for assessing its exact location, size, and relationship with adjacent structures, which helps guide curative resection. Moreover, imaging modalities such as EUS and MRI can aid in ruling out metastatic disease or involvement of critical vascular structures [21,22].

In our case, the histopathological examination revealed that the tumor is well-differentiated. Well-differentiated NETs typically display uniform, small- to medium-sized cells arranged in an organoid pattern, often forming nests or a trabecular or gyriform pattern. The tumor cells have eosinophilic, finely granular cytoplasm due to the abundance of neurosecretory granules, with centrally located, round, and uniform nuclei. The chromatin shows a finely stippled salt-and-pepper appearance, and the nucleoli are either inconspicuous or absent. Mitotic figures are rare, and necrosis is typically absent. Immunohistochemical staining for specific hormones in a primary tumor is generally recommended only when a clinical hormonal syndrome is present. Functionality is determined by the presence of symptoms rather than positive immunohistochemical staining for specific hormones. Therefore, positive insulin staining is not obligatory for diagnosing insulinomas and is typically not required once the clinical diagnosis has been established [25]. Chromogranin A, a protein integral to the membrane of neurosecretory granules, is expressed in the majority of NETs, while synaptophysin, a protein component of the small synaptic vesicles, is present in all neuroendocrine cells. Immunohistochemical staining for the mitotic index using Ki-67 provides essential prognostic information. The Ki-67 staining rate in metastastic tumor tends to correlate with that in the primary tumor and can be used for tumor grading [20]. However, in some cases, heterogeneity may occur, and patients with variations in the Ki-67 index between primary and metastatic sites tend to have a poorer prognosis [26]. Ki-67 is consistently recognized as a key predictor of tumor behavior and is included in the WHO classification [25]. A previous study also demonstrated that a Ki-67 index above 2% is associated with significantly higher mortality, highlighting its importance in predicting patient outcomes [27].

Two commonly used staging systems for P-NETs have been established, one by the European Neuroendocrine Tumor Society (ENETS) and the other by the American Joint Committee on Cancer (AJCC). Tumor grading, differentiation, and staging are important in determining the appropriate treatment for patients with P-NETs. Treatment options for P-NETs include active surveillance with periodic imaging follow-up, surgical resection, chemotherapy, and radiation therapy. For well-differentiated grade 1 and grade 2 P-NETs in the early stages, complete surgical resection with regional lymph node dissection is the treatment of choice and should be considered for all patients with early stage disease. In small (<1.5 cm) low-grade non-functioning P-NETs, conservative management with follow-up imaging may be an option. However, surgical resection remains the preferred choice for functioning and larger non-functioning P-NETs whenever feasible, which is the treatment option selected for our case of small and functioning insulinoma. Aggressive surgical resection and tumor debulking are important for symptomatic patients with advanced metastatic disease. Following surgical intervention, insulinomas are typically curable for most patients. In cases where surgical resection is not feasible, medications such as diazoxide and octreotide may be used [22,28].

## Conclusions

Insulinoma, although uncommon and typically benign, can present with symptoms that are easily overlooked, leading to delays in diagnosis and potentially escalating to life-threatening hypoglycemic episodes. While biochemical diagnosis is straightforward, the initial recognition and preoperative localization of the tumor can be challenging. Early recognition of insulinoma symptoms requires increased clinical awareness of nonspecific signs such as episodic confusion, sweating, or palpitations, particularly in non-diabetic individuals. A detailed patient history, including Whipple's triad, should prompt further investigation. EUS with FNA might detect small lesions, particularly those deep within the pancreas. CT with contrast identifies small hypervascular lesions, while MRI provides superior soft tissue contrast to differentiate the lesion from surrounding tissue. Surgical resection remains the treatment of choice with an exceptionally high success rate. Clinicians should maintain a heightened level of suspicion for insulinoma, particularly in patients with a history of recurring episodes of hypoglycemia.
